# Overexpression of MADS-box Gene *AGAMOUS-LIKE 12* Activates Root Development in *Juglans sp.* and *Arabidopsis thaliana*

**DOI:** 10.3390/plants9040444

**Published:** 2020-04-02

**Authors:** Grégory Montiel, Muriel Gaudet, Françoise Laurans, Philippe Rozenberg, Matthieu Simon, Pascal Gantet, Christian Jay-Allemand, Christian Breton

**Affiliations:** 1 INRAE Val de Loire–Orléans, UMR 0588 BioForA INRAE-ONF, 2163 avenue de la pomme de pin, CS 40001 Ardon, CEDEX 02, 45075 Orléans, France; gregory.montiel@univ-nantes.fr (G.M.); murielvirginie.gaudet@cnr.it (M.G.); francoise.laurans@inrae.fr (F.L.); philippe.rozenberg@inrae.fr (P.R.); 2 Laboratoire de Biologie et Pathologie Végétales (EA 1157), 2 rue de la Houssinière, BP 92208, 44322 Nantes, France; 3 National Research Council (CNR), Institute of Research on Terrestrial Ecosystems (IRET), Via G. Marconi N. 2, 05010 Porano (TR), Italy; 4 Institut Jean-Pierre Bourgin, INRAE-AgroParisTech, UMR1318, Bâtiment 7, INRAE Centre de Versailles-Grignon, Route de St-Cyr, CEDEX, 78026 Versailles, France; matthieu.simon@inrae.fr; 5 Université de Montpellier, UMR DIADE, 911 avenue Agropolis, CEDEX 05, 34394 Montpellier, France; pascal.gantet@umontpellier.fr; 6 Université de Montpellier, UMR IATE (UM, INRAE, CIRAD, SupAgro), CC024, Place Eugène Bataillon, CEDEX 05, 34095 Montpellier, France; christian.jay-allemand@umontpellier.fr

**Keywords:** transgenic plant, root meristem, cell division, cell differentiation, transcription factor

## Abstract

Until recently, the roles of plant MADS-box genes have mainly been characterized during inflorescence and flower differentiation. In order to precise the roles of *AGAMOUS-LIKE 12*, one of the few MADS-box genes preferentially expressed in roots, we placed its cDNA under the control of the double 35S *CaMV* promoter to produce transgenic walnut tree and *Arabidopsis* plants. In *Juglans sp.*, transgenic somatic embryos showed significantly higher germination rates but abnormal development of their shoot apex prevented their conversion into plants. In addition, a wide range of developmental abnormalities corresponding to ectopic root-like structures affected the transgenic lines suggesting partial reorientations of the embryonic program toward root differentiation. In *Arabidopsis*, *AtAGL12* overexpression lead to the production of faster growing plants presenting dramatically wider and shorter root phenotypes linked to increased meristematic cell numbers within the root apex. In the upper part of the roots, abnormal cell divisions patterns within the pericycle layer generated large ectopic cell masses that did not prevent plants to grow. Taken together, our results confirm in both species that *AGL12* positively regulates root meristem cell division and promotes overall root vascular tissue formation. Genetic engineering of *AGL12* expression levels could be useful to modulate root architecture and development.

## 1. Introduction

Since the discovery in the early nineties of floral homeotic mutants characterized by drastic developmental changes (i.e., ectopic organ formation), MADS-box genes whose mutations generated such variants during floral organ differentiation in model species like *Arabidopsis*, *Petunia* or *Antirrhinum* have become one of the most studied gene family in the plant kingdom [1,2,3]. Found in every eukaryote from yeasts to animals, expression and phylogenetic studies revealed a prominent expansion of the MADS-box gene family in plant genomes but also highlighted several clades of these transcription factor (TF) as being preferentially expressed in vegetative structures, widening the range of their roles during plant development [4,5,6,7]. Northern-blots performed with a small set of MADS-box cDNAs showed that *AGL12*, *AGL14* and *AGL17* were preferentially expressed in *Arabidopsis* roots suggesting possible roles for these transcription factors in the development of this organ [8]. *ANR1* was the first MADS-box gene shown to be involved in root development by mediating lateral root formation in response to nitrate presence [9]. Later, *AGL14* was reported to regulate auxin transport in the root through direct regulation of *PIN1* and *PIN4* auxin transporter genes and *AGL21* shown to integrate various stress-related nutritional and hormonal stimuli into auxin signals required for lateral root primordium initiation and outgrowth [10,11]. Hence, MADS-box genes may be, through their diversity and redundancy, one the primary TF family to regulate various plant organogenesis programs, plant stress responses and plasticity by integrating environmental and hormonal signals [12,13,14]. Deciphering the gene regulatory networks (GRN) controlled by such master genes should provide a better understanding of various developmental programs of great importance not only in model species but also in cultivated species and trees.

According to the current climate change predictions, the adaptation and survival abilities of long living trees are clearly expected to be challenged by increasing numbers of threats (drought periods, floods, storms, exogenous pest invasions…). As flower and seed sets seem to be already affected in currently harvested seed orchards by recurrent heat wave and/or drought periods [15,16], the genetic cues controlling root development and root architecture could represent important factors to consider in tree breeding and selection programs. Within this framework, *AGL12/Xal1* appeared as a candidate of choice to initiate such transfer from *Arabidopsis* to one of our species of interest (walnut tree) in order to evaluate its potential usefulness as starting point for molecular physiology studies of root development in trees. Indeed, the *AGL12* clade is one of the rare single gene branch of the complete *Arabidopsis thaliana* MADS-box genes phylogenetic tree [17,18]. In situ mRNA hybridization and promoter::reporter gene fusions showed that *AGL12* was mainly expressed in the central cylinder of mature roots and in the lateral root primordia but also presented punctual expression patterns in the primary root meristem, the atrichoblasts of the root epidermis as well as in developing embryos and flowers [19,20]. Mutant allele phenotype observations and computational analysis of microarray datasets highlighted some roles of this TF in the regulation of root development, flowering time and secondary cell wall formation [21]. Finally, *AGL12/XAL1* was recently shown to be an important regulator of cell proliferation within the *Arabidopsis* root meristem. *Xal1* mutants presented lower cell division rates, abnormal periclinal divisions in the quiescent center and deformed columella cells leading to short-root phenotypes. Conversely, *XAL1* overexpression under the 35S promoter complemented *xal1* mutants and resulted in an increase of both root meristem size and meristematic cell number in transgenic plants through a positive regulation of *PLETHORA 1* (*PLT1*) and other important cell cycle genes [22].

Since the functions of the MADS-box genes controlling flower development had mainly been determined through the study of loss or gain of function mutants, we used the same strategy to investigate the roles of *AGL12* in root development and potentially modify the development and/or architecture of this organ. In the present study, we placed the *Arabidopsis thaliana AGL12* cDNA (*AtAGL12*) sequence under the control of the double 35S Cauliflower Mosaic Virus constitutive promoter (d35S) in order to transform walnut tree and *Arabidopsis thaliana.* Using both sense and antisense construct associated to this stronger promoter well-adapted to the study of transgenic T_1_ in poplar and walnut tree [23,24,25], we show here that up and down modifications of *AGL12* expression have significant developmental effects in both of these heterologous and homologous recipient species. In *Juglans sp.*, overexpression of *AtAGL12* increased significantly the germination rates of the transgenic somatic embryos. In addition, morphological aberrations corresponding to early and partial stages of ectopic root development were regularly observed among the transgenic lines overexpressing the transgene. In *Arabidopsis*, shorter but thicker roots were systematically observed in T_1_ plantlets overexpressing *AtAGL12*. These phenotypes were linked to increased cell division and cell differentiation patterns within the root apical meristem (RAM) and other reactive parts of the roots while shoot apical development was positively affected. Taken together, our results suggest that *AGL12* represents an interesting candidate to study and engineer root architecture in trees whose root systems remain very difficult to access for developmental studies and phenotyping. 

## 2. Results

### 2.1. Walnut Tree Transformation

#### 2.1.1. Walnut Tree Somatic Embryo Transformation

**Figure 1:**
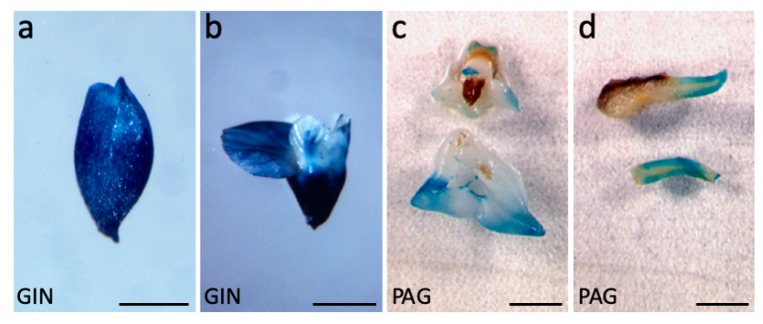
*Walnut tree somatic embryo transformation.* (**a**) Expression of d35S::*GUS* in a transgenic early cotyledonary stage walnut somatic embryo (GIN5 line). (**b**) Expression of d35S::*GUS* in a mature GIN1 somatic embryo. (**c**) Patchy expression of *pAtAGL12::GUS* in the cotyledons of mature somatic embryo (PAG75 line). (**d**) Expression of *pAtAGL12::GUS* in the root of a germinated somatic embryo (PAG15 line). Scale bars: 2.5 mm in (**a**); 5 mm in (**b**–**d**).

In our hands, up to 75% of false positives had been routinely observed in previous walnut transformation experiments [25]. We first used the *d35S::GUS-intron* construct to optimize hybrid walnut tree transformation and visualize the expression patterns of the d35S promoter used to drive *AtAGL12* expression in walnut somatic embryos. Sixty to 110 kan^R^ secondary embryos could be isolated from each batch of primary somatic embryos subjected to agrobacteria infection. Addition of sand during the inoculation step increased by 10% the proportion of *GUS*^+^ secondary embryos. The number of false positives was also significantly reduced by increasing the number of subcultures on selection medium from three to five. In the end, 45% of the established GIN embryonic lines showed intense β-glucuronidase activities in every part of the developing embryos (Figure 1a). In mature embryos, the root apical poles of the embryos were intensively colored (Figure 1b).

PAG, S, and AS embryonic lines were obtained through this optimized protocol. Walnut tree somatic embryo transformation and transgenic line selection results are summarized in Table 1. Out of about one hundred transgenic lines analyzed, a great majority presented 1 to 5 T-DNA insertion events revealed by Southern-dot- and Southern- blot analysis. Only a few lines showed more complex patterns involving ten or more insertions (not shown).

#### 2.1.2. The Promoter of AtAGL12 is Functional in Walnut Tree Somatic Embryos

The activity of the *Arabidopsis thaliana AGL12* promoter fused to the *GUS* reporter gene was investigated during walnut tree somatic embryo development (PAG lines). In well-developed transgenic embryos, only punctual β-glucuronidase activities were regularly observed in the cotyledons and the outer tissues of the embryonic root (Figure 1b,c). Once converted into plantlets, GUS activities could not be observed due to instant and massive darkening of the root tissues in the reaction solution (not shown). These strong oxidation reactions could be linked to important flavonoid and naphtoquinone accumulation in developing roots in this species [26,27].

#### 2.1.3. AtAGL12 Activates the Germination of Walnut Tree Somatic Embryos

**Figure 2:**
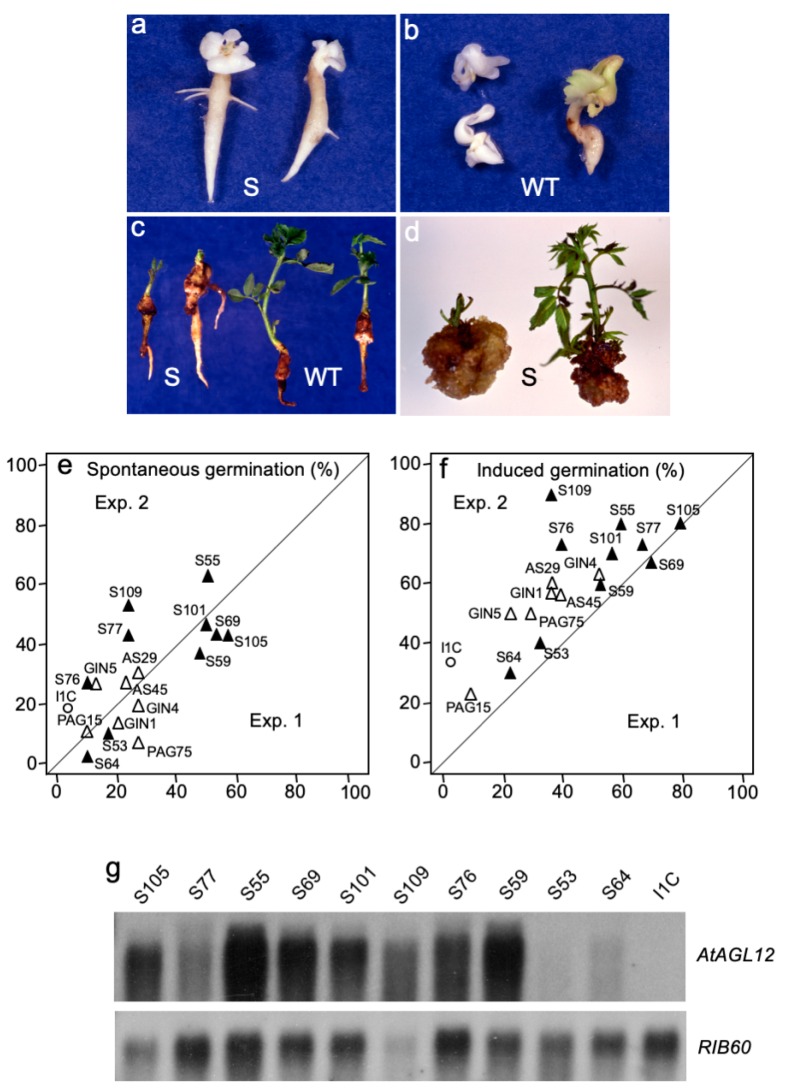
*AtAGL12* overexpression in walnut somatic embryos and plantlets. (**a**) Accelerated spontaneous germination of S101 somatic embryos overexpressing *AtAGL12* in sense orientation after 3 weeks of horizontal culture. (**b**) Wild-type I1C embryos sampled at the same time as control. Note the earlier initiation of lateral root development among the S embryos; (**c**) conversion of walnut somatic embryos into plantlets. S109 transgenic plantlets (left) and wild type I1C control (right) were photographed after 5 weeks of development in individual tubes. Note the much more important root to shoot ratios for the S plantlets; (**d**) variable micropropagation and shoot development abilities of the transgenic S lines: the S69 line (left) presenting high *AtAGL12* expression levels is characterized by poor shoot apical development and important callus development. Conversely, the S64 line (right) characterized by a low transgene expression levels presents a normal shoot apical development; (**e**) spontaneous germination rates of representative walnut somatic embryo S, AS, PAG, GIN and wild type I1C lines were scored after 3 weeks of culture. For each line, the numbers of germinated somatic embryos were determined twice (experiment 1 and 2, n = 3 × 10). (**f**) Induced germination rates were scored after the ablation of the cotyledons and 2 additional weeks of vertical culture (for a total of five weeks of culture). The average germination percentages observed for each S (closed triangles, bold characters), transgenic control (AS, GIN and PAG lines, open triangles) and the wild-type I1C lines (open circle, bold characters) are reported for both experimental repeats and culture times; (**g**) relative expression levels of *AtAGL12* detected in mature non-germinated embryos of the selected walnut transgenic S lines revealed by northern-blot. A walnut *Rib60S* cDNA probe was used as reference.

We then addressed the roles of *AGL12* on root development by overexpressing its cDNA in sense (S) or antisense (AS) orientations in walnut tree. The *AtAGL12* S and AS constructs were used to generate transgenic walnut tree somatic embryo lines (Figure 2). About 30 S and AS kan^R^ lines were initially selected in order to be sub-cultured along with the wild-type I1C line and the other controls (GIN and PAG lines). Observations performed on these lines over a six months period (10 to 12 subcultures) revealed that somatic embryos of the S55, S59, S69, S77, S101 and S105 lines (Figure 2a) presented relatively higher spontaneous germination rates than those of the GIN, PAG AS and WT embryos (Figure 2b). Hence, ten representative S lines (Table 1) presenting high, medium and low germination abilities were selected for further analysis and confirm the effects of *AtAGL12* overexpression on the development of the embryonic root. 

In vitro spontaneous and induced germination abilities of these lines were determined after 3 and 5 weeks of culture and compared to those of the WT, GIN, PAG and AS control lines (Figure 2e,f). At both stages, the germination and development abilities of the AS embryos did not differ significantly from those of the I1C, GIN and PAG lines (Student’s test, 0.15 < *p* < 0.94) suggesting no evident interferences between the AS *AtAGL12* and the endogenous *AGL12* mRNAs in this recipient species (Supplemental Figure S1). After 3 weeks of culture, the S lines showed significantly higher germination rates visualized by the spontaneous development of primary embryonic roots (Figure 2e–g). At this stage, the embryos of the S lines had germinated at an average of 33.5% ± 5.2% compared to 12.5% ± 11.6% and 15.7 ± 3.2% for the I1C and the other transgenic control lines (AS, PAG and GIN) (Student’s test, *p* = 0.007 and 2.6 × 10^−7^, respectively). Higher germination rates were also observed for the S embryos after being submitted for 2 additional weeks to a standard germination induction protocol including the excision of the cotyledons and vertical growth of the embryonic axis (Figure 2f). At the end of the fifth week of culture, 59.2% ± 5.8% of the embryos of the S lines germinated whereas only 22.5% ± 16.0% of the I1C embryos and 39.0% ± 6.9% of the transgenic controls developed roots (Student’s test, *p* = 7.1 × 10^−5^ and 2.3 × 10^−5^, respectively). Among the S lines, S64 and S53 presented systematically the lowest germination rates whereas S77 and S105 peaked with 70 to 80% of root production. 

Lateral roots seemed to appear faster among the S embryos (Figure 2a). However, even if 10.2% ± 4.1% of the primary roots that had grown after 3 weeks of culture showed at least one lateral root for these lines, this value was not significantly different from those observed for the I1C (4.0% ± 4.9%, Student’s *t* test, *p* = 0.4) and the transgenic control lines (5.6% ± 4.5%, Student’s test, *p* = 0.2). Conversely after 5 weeks, significantly higher percentage of I1C germinated embryos presented lateral roots (56.7% ± 29.1%) compared to the S (22.7% ± 5.2%, significantly different, Student’s *t* test, *p* = 0.002) or the other transgenic embryos (18.7% ± 6.8%, significantly different, Student’s *t* test, *p* = 0.006). This tendency was confirmed at the end of the fifth weeks of the germination experiments as the number of lateral roots produced per rooted embryo was significantly higher for the I1C embryos (3.6% ± 1.1%) compared to the S (2.2% ± 0.2%, Student’s *t* test, *p* = 0.008) or the other transgenic embryos (2.2% ± 0.3%, Student’s *t* test, *p* = 0.03). 

The development of the plantlets obtained after somatic embryo conversion was further analyzed for the different lines. After a month and a half of culture, the root systems of the S plantlets appeared to be more developed while their shoots were shorter and their leaves atrophied (Figure 2c). This asymmetric development was confirmed by dry weight measurements and the calculation of individual root/shoot ratios (Table 2). Reduced shoot development among the S lines presenting moderate to high levels of transgene expression was also observed during classical micropropagation (Figure 2d). In the end, no plant could ever be recovered and transferred in the greenhouse for any of the S lines.

In accordance with a walnut (*wAGL12*, accession number MF327581) and *Arabidopsis AGL12* cDNA sequences comparison (Supplemental Figure S1), *AthAGL12*/*wAGL12* cross hybridizations or probing I1C walnut genomic DNA with *AtAGL12* cDNA did not reveal any hybridization pattern even at low stringency. Hence, transgene expression levels were specifically detected by northern-blot in each of the phenotypically selected S lines (Figure 2g). Embryos of the S64 and S53 lines showed the lowest relative expression levels of *AtAGL12* while those of the S55, S105, S59 and S109 presented the highest. Overall, the intensity of hybridization correlated well with the germination rates observed for the S lines (Figure 2g) suggesting that the biological effects observed on the rooting and developmental abilities of these lines (i.e., spontaneous germination, shoot atrophy) are effectively linked to *AtAGL12* overexpression. Conversely, antisense overexpression of *AtAGL12* did not affect the development of walnut AS embryogenic lines as its sequence did not apparently interfere with endogenous *AGL12* mRNAs (Supplemental Figure S1).

#### 2.1.4. AtAGL12 Overexpression Causes Ectopic Root-like Structure at Different Stages of Walnut Tree Somatic Embryogenesis

**Figure 3:**
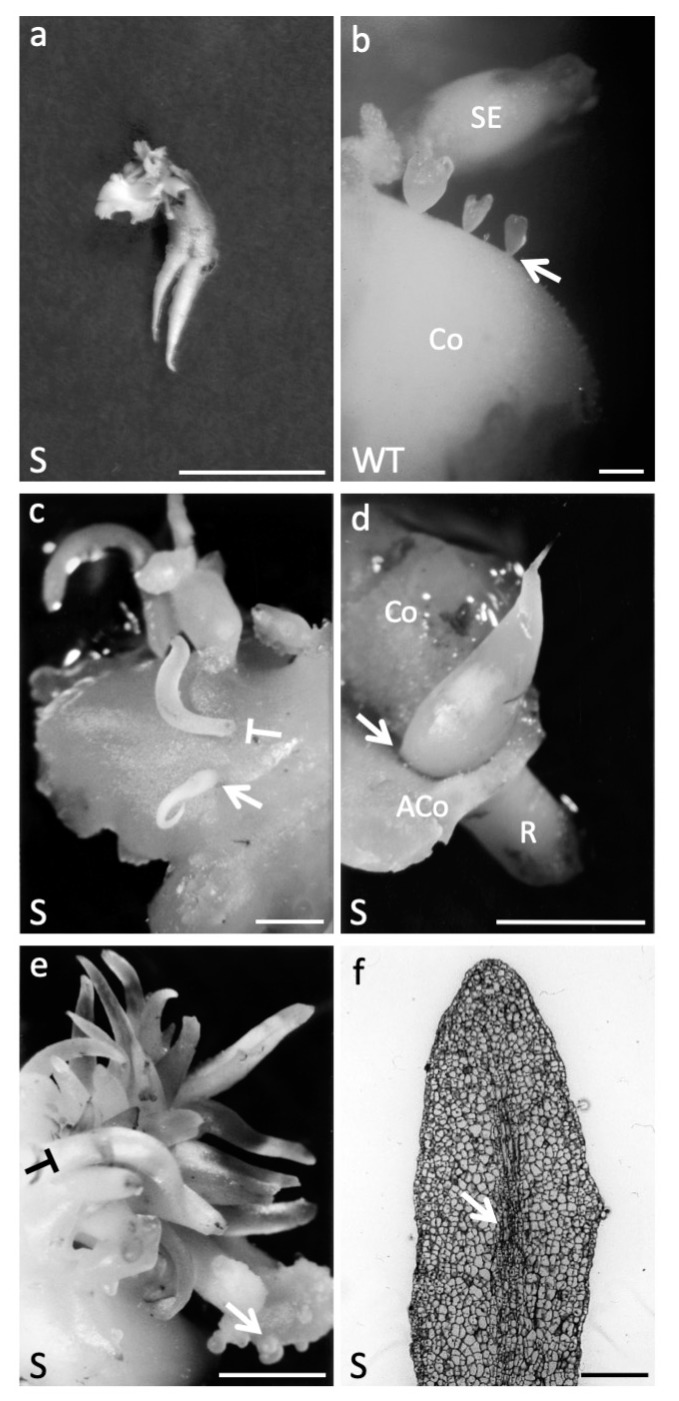
Developmental abnormalities linked to *AtAGL12* overexpression in walnut. (**a**) S77 somatic embryo (SE) showing two fused primary roots after 3 weeks of culture. SE presenting fused primary roots were only observed among S embryogenic lines. Scale bar: 1 cm; (**b**) normal repetitive secondary somatic embryogenesis process in wild-type walnut tree I1C line. The picture shows different stages of SE emerging from the cotyledon (Co) of a primary embryo. The arrow points to the thin basal parts of the secondary embryos that develop from single activated epidermal cells. Scale bar: 1 mm. (**c**) Ectopic root-like structure (arrow) developing on the apical side of a globular embryo (S77) in place of a regular shoot apex. No cotyledon will form. As in 1b, note the small size of the base of the embryo linked to its single cell origin within the primary cotyledon’s epidermis. When such root-like structures develop directly from activated cells within the epidermal layer, they present a thicker base and need to be cut of the cotyledon in order to be separated from it (T-shaped symbol as in 1e). Scale bar: 0.5 cm; (**d**) root-like structure developing on the apical part of mature S77 somatic embryo. Excision of the cotyledon reveals the base of the structure originating at the cotyledon/shoot apical meristem junction (arrow). Co: cotyledon, ACo: ablated cotyledon, R: embryonic root pole. Scale bar: 1 cm; (**e**) cluster of ectopic root-like structures emerging from activated epidermal cells (S76). Clusters of secondary somatic embryos can be observed on the same initial explant (arrow). Scale bar: 0,5 cm; (**f**) longitudinal section of an ectopic root-like structure (S76). In the central axis of these structures one can observe the initiation of formation of a vascular bundle (arrow) in the absence of a clearly defined apical meristematic zone. Scale bar: 500 μm.

In addition to increased germination abilities, some unique developmental characteristics could be observed on a regular basis during the propagation and the maintenance of the S embryonic lines (Figure 3). First, compared to normally germinating WT I1C embryos (Figure 2b), some germinating S embryos were characterized by the development of multiple primary roots (Figure 3a). Fourteen of these cases were precisely accounted for during the germination experiments involving 600 S and control embryos (I1C, GIN, PAG and AS), respectively. Supplementary roots were only observed among the S55, S69, S76, S77, S101, S105 and S109 lines and up to four roots (fused or not) could be counted per embryos. The second kind of developmental abnormalities were observed throughout S somatic embryo development. The normal repetitive secondary embryogenic process allowing the indefinite in vitro maintenance and propagation of the WT I1C embryonic line is presented in Figure 3b. Commonly observed among the S embryos, these abnormalities corresponded to ectopic root-like structures developing on the shoot apical part of immature or mature somatic embryos (Figure 3c,d, respectively). Finally, such structures were also and much more frequently observed on the surface of developing cotyledons where they grew in clusters that could cover up a half cm^2^ (Figure 3e). Histological observations of these outgrowing structures showed important similarities with a normal root apex with the exception that they lacked well-defined meristematic zones at their tips (Figure 3f). Indeed, we never managed to get these structures to differentiate further into well-formed roots after being dissected and directly sub-cultured on DKW medium supplemented with various growth regulators. 

### 2.2. AtAGL12 Activates Cell Division and Differentiation in Arabidopsis thaliana Root Meristem

**Figure 4:**
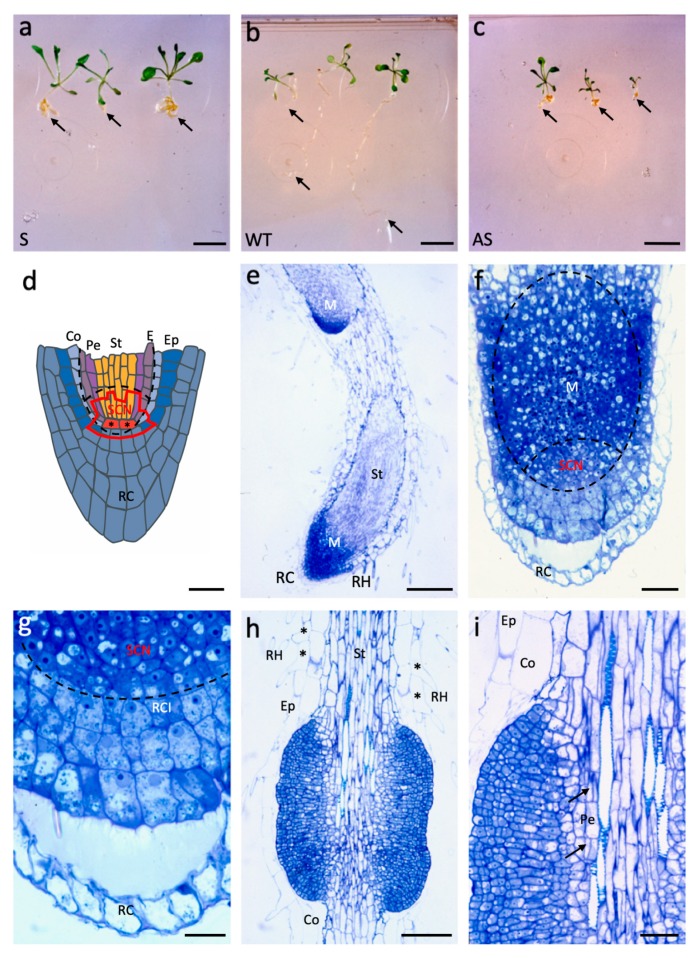
*AtAGL12* overexpression in *Arabidopsis thaliana*. (**a**) Sense (S) T_1_ kan^R^ plantlets 10 days after germination; scale bar: 1 cm; (**b**) wild-type (WT) plantlets 10 days after germination; scale bar: 1 cm (**c**) Antisense (AS) T_1_ kan^R^ plantlets 10 days after germination; scale bar: 1 cm; for each plantlet, an arrow points to the apical root tip. (**d**) Schematic longitudinal section of a WT *Arabidopsis* root tip adapted from [28]. The main cell types forming the root apical meristem are indicated in different colors: RC: root cap (grey-blue); Ep: epidermis (dark blue); Co: cortex (light blue); E: endodermis (purple-grey), Pe: pericycle (purple); St: Stele (orange). In the stem cell niche (SCN, encircled in red) of the meristem, the quiescent center cells (QC, red with asterisks) are surrounded by a small number of initial cells whose descendants will further divide and differentiate. The main areas of the meristem affected by *AtAGL12* overexpression are encircled by dashed lines (see 1f and 1g). Scale bar: 20 μm. (**e**) Longitudinal section of the primary root tip of a S plantlet presenting two fused roots. In S plantlets, the apical root meristematic zone (M) is enlarged and the stele (St) wider. RH: root hair. Scale bar: 100 μm. (**f**). Longitudinal section of a S plantlet root tip. The meristematic areas principally affected by *AtAGL12* overexpression are encircled (dashed lines). Compared to WT (1d), the basal SNC area of the meristem is wider, characterized by a greater number of meristematic cells. Scale bar: 20 μm. (**g**) Closer view of the 1f root tip focused on the SNC area. Scale bar: 10 μm; (**h**) longitudinal section of the upper part of the root of a S plantlet showing numerous cell divisions resulting in the formation of abnormal cell masses around the stele. The presence of adjacent trichoblasts (*) developing root hairs (RH) can be observed. Ep: epidermis, Co: cortex, St: stele. Scale bar: 50 μm. (**i**) Closer view of the cellular protuberance. The arrows point to closely occurring periclinal cell divisions within the pericycle layer (Pe). Further, both anticlinal and periclinal cell divisions explain the important outgrowth of these structures. Scale bar: 20 μm.

Since *AtAGL12* overexpression led to dramatic developmental and morphological modifications of walnut somatic embryogenesis, the same constructs were used to transform *Arabidopsis* (Figure 4). In this homologous transgene recipient system, both S and AS constructs were expected to be effective. Indeed, upon germination every kan^R^ T_1_ S seedlings (Figure 4a) grew larger and presented much shorter but thicker roots than WT controls (Figure 4b). Conversely, AS plantlets (Figure 4c) were smaller, developed slowly, and presented small root systems suggesting successful interferences between the overexpressed AS sequence and endogenous *AGL12* mRNAs.

Due to the important changes affecting the sizes of both the roots and the apical parts of S plantlets whose transfer could be of major interest in trees, we focused our analysis on positively affected S transgenic plants. Three S and WT plantlets were sampled for histological comparisons while every other S and AS T_1_ were transferred to the greenhouse for T_2_ production and further molecular and phenotypic characterizations. In our hands, WT roots were too fine to obtain any satisfactory longitudinal sections of their apex but cross sections of WT and S roots are presented in Supplemental Figure S2. Yet, compared to a schematic longitudinal representation of the well-described WT *Arabidopsis* root apex (Figure 4d, adapted from [28]), longitudinal sections of S roots provide clear views of the dramatic changes induced by *AtAGL12* overexpression in T_1_ plantlets (Figure 4e,f).

At low magnification, observations of the primary roots of 3 weeks old S plantlets revealed drastically enlarged RAM leading to an important increase of the number of cell rows forming the stele (Figure 4e,f). In S plantlets, this number varied between 17 to 32 on the two types of sections observed (transversal and longitudinal) whereas only eight to 10 cells were recorded in WT transverse sections (Supplemental Figure S2). The numbers of cell layers forming the outer tissues of the S roots (i.e., cortex and epidermis) did not appear to be affected by *AtAGL12* overexpression. Closer observations of S root tips revealed important and unorganized cell division patterns in the basal part of the meristem leading to less defined cell lineage patterns in its upper part corresponding principally to the stele differentiating zone (Figure 4f). Whereas the root cap does not appear to be affected in S plants, the cells within the SCN area are small, present dense cytoplasm and small vacuoles related to their meristematic status (Figure 4g). Interestingly, increased cell division activity was also observed in the upper part of S roots leading locally to the formation of ectopic cell masses around the stele (Figure 4h). In these structures, the most internal cell divisions patterns were localized within the pericycle cell layer of the root (Figure 4i). Thereafter, periclinal and anticlinal cell divisions seem to be involved in their expansion. These overgrowing structures could correspond to enlarged or merging meristematic areas linked to *AtAGL12* overexpression that might affect the early stages of lateral root formation in the upper part of the roots. In this part of the roots, slighter alterations could also be noticed within the epidermis layer where adjacent trichoblats developing root hairs where regularly observed (Figure 4h).

In the end, both of these major structural variations affecting the root apical meristem and the upper part of the roots did not prevent the growth of the S plantlets. Conversely, S plants grew larger during the whole in vitro culture period (Figure 4a). In the greenhouse, S plants continued to grow faster than WT while AS plants remained much smaller. Both S and AS plants produced flowers and seeds but no differences could be observed between T_2_ S, AS transgenic lines and WT suggesting a systematic post-transcriptional repression of transgene expression in homozygous plants.

## 3. Discussion

Because of their relatively simple structure consisting of three main concentric tissue layers (epidermis, cortex and stele), roots represent an attractive model to study cell fate and cell differentiation within a single organ [29]. As for shoots, root development results from the activation of intricate morphogenetic programs that are mainly unraveled through studies performed on model species *Arabidopsis thaliana*. GRN governing the establishment of the primary and lateral root meristems, the radial tissue patterning and the basal apical differentiation axis have been analyzed through the characterization of important TFs and the study of developmental mutants, many of them being linked to hormonal and nutrient metabolism [30,31,32,33]. As a potential target to increase plant biomass production or adaptation abilities, root development has already been subjected to genetic engineering [34]. However, many of these attempts lead concomitantly to important reductions of shoot growth [35].

In this work, we prospected the use of MADS-box genes to modify root development and analyze their potential roles during the growth of this organ. *AGL12*, present as a single clade member preferentially expressed in *Arabidopsis* roots, appeared to be an attractive candidate for these purposes. Indeed, *AGL12/XAL1* had already been shown to be an important regulator of cell proliferation in *Arabidopdis* root apex [21,22] and we had previously shown that the overexpression of *AtAGL12* promoted the aggregation of *Catharantus roseus* suspension cells into parenchyma-like structures and the production of alkaloids [36]. In the present study, we used the same strategy to overexpress *AtAGL12* cDNA in sense and antisense orientations in heterologous (*Juglans sp.*) and homologous (*A. thaliana*) recipients in order to amplify some of its effects on root development.

In *Juglans sp.*, high expression levels of *AtAGL12* correlate well with increased germination abilities of the S embryos. Our results suggest that an expression threshold of this single heterologous TF appears to be sufficient to activate cell division in both apical embryonic meristems leading to an increased root biomass production but also to a disorganization of the shoot apex meristem preventing the production of any plantlet (Figure 2c). This phenomenon was observed in most transformation experiments aimed at improving root development [35]. In our walnut S lines, the shoot apical disturbances might be explained by inefficient post-transcriptional regulation process of the transgene (i.e., miRNAs) since the overexpressed heterologous *AtAGL12* would not present enough sequence similarities with the corresponding endogene (Supplemental Figure S1). Indeed, such apical abnormalities were not observed in *Arabidopsis* S T_1_ plantlets (Figure 4a) in which apical post-transcriptional regulation of the overexpressed homologous transgene sequence would be effective. Upon germination, the roots of the S walnut somatic embryos seemed to grow faster as visualized by the precocious appearance of lateral roots in some lines (Figure 2a). However, the data collected overall on the selected S lines showed that the differences concerning lateral root development were not significant after 3 weeks of germination and even reversed after 5 weeks. This result could be explained by a progressive reduction of root growth rates due to the lack of shoot development or to environmental competition since much more S embryos had germinated in each Petri dish compared to the wild-type. Finally, another explanation would imply a negative effect of *AtAGL12* expression on lateral root initiation. To test these possibilities and avoid the negative effects of *AtAGL12* constitutive overexpression in the shoot apex, we tried to graft in vitro wild-type shoot tips explants onto S line rootstocks in order to produce chimeric plants and study their development in the greenhouse [37]. Unfortunately, walnut tree micrografting was unsuccessful in our hand. However, the use of genetically engineered rootstock for enhanced *AGL12* expression could be transferred to other “transformable” horticultural species or poplar. In the end, the use of root-specific or artificially inducible promoters could also provide interesting perspectives to specifically alter *AGL12* expression levels in roots without any major disturbances of the shoot apical part. The development of such transgenic plants or trees could provide interesting models to study the impacts of the root systems on biomass production, carbon allocation, shoot/root ratios as well as on drought or wind resistance in the general context of climate change.

Interestingly, numerous developmental abnormalities corresponding to the development of root-like structures were observed in the transgenic *Juglans sp.* somatic embryo lines overexpressing *AtAGL12*. Their formation at different places on the embryos or cotyledons appears coherent with the d35S expression patterns observed throughout walnut somatic embryo development (Figure 1a,b) and may be explained by local cell division activations at different stages of the secondary embryogenesis process and partial reorientations of this developmental program toward root development. In *Juglans sp.* normal secondary embryogenesis process, clusters of young somatic embryos arise through perpendicular divisions of single cotyledonary epidermal cells (Figure 3b) [38]. In S lines, the overexpression patterns of *AtAGL12* placed under the control of the d35S promoter may interfere locally with these morphogenetically activated surface cells to deviate their development from the embryonic program toward the production of root-like structures well-anchored to the cotyledon (Figure 3c,e). Then, during early embryo development, the same type of morphogenetic interferences could affect the apex of globular stage embryos to induce the formation of pointy embryos that consequently, as regular somatic embryos, are easy to separate from the cotyledon they originated from (Figure 3c). Later, a partial reprogramming of meristematic cells at the cotyledon/embryo axis junction would explain the formation of the ectopic structures developing on the apex of more mature transgenic embryos (Figure 3d). Despite their important structural resemblance to roots, the development of these ectopic structures was never sufficiently reoriented to lead to the formation of a functional root meristem (Figure 3f) as observed for *PLETHORA* mutants [39]. Indeed, the only supplementary functional roots that developed in the S walnut tree embryogenic lines originated from developmentally determined root apex of germinating transgenic S embryos (Figure 3a). Thus, the overexpression of *AtAGL12* in *Juglans sp.* does not appear to allow by itself a complete homeotic conversion of embryogenetically programmed cells into functional root apex but rather induce the activation of cell division and partial cell differentiation process in meristematic or developmentally activated cells. Indeed, both of these roles were confirmed in *Arabidopsis thaliana* transgenic plants.

In *Arabidopsis*, the ectopic overexpression of *AtAGL12* resulted also in majorly altered phenotypes for both S and AS T_1_ transgenic plantlets (Figure 4). AS plantlets (Figure 4c) presented much shorter roots and were smaller than the wild-type plants (Figure 4b). These phenotypes are consistent with the Xal1/AGL12 mutants described in [20]. Indeed, antisense RNA or knock-out mutations are more likely to affect the expression of genes present as single copy in the genome like *AGL12*. Conversely, S plantlets (Figure 4a) grew faster and produced larger leaves while their roots were shorter but much thicker due to important activations of cell division both in the root apical meristem and in the upper parts of the root. At both locations, the induced cell divisions lead to major increases in cell number within the reactive meristematic structures (Figure 4e–i). These observations are in accordance with previous results concluding that *AGL12* was a strong regulator of cell division in *Arabidopsis* roots and *C. roseus* cultured cells [20,36]. In the end, the enhanced apical shoot growth observed in the *Arabidopsis* S plants might be explained by an indirect effect of *AtAGL12* overexpression in the roots whose enlarged vascular bundle may improve nutrient transfer to the shoot or by direct activation of cell divisions within the shoot and leaf tissues. *AGL12/XAL1* has recently been shown to activate the expression of *Plethora 1* (*PLT1*) and other important cell cycle genes in the root meristem [22]. Since *AGL42* has been shown to be a specific marker of the quiescent center [40], one could also assume that the overexpression of *AtAGL12* could eventually interfere with this MADS-box TF or lead to its replacement within the MADS-box transcription regulatory complexes formed in this particular area of the transgenic roots. Interestingly, transgenic lines overexpressing *AGL12*/*XAL1* under the 35S promoter showed delayed cell differentiation as their root hairs appeared further away from the root tip [22]. In our study based on the use of d35S based constructs, quite opposite phenotypes are obtained (Figure 4). S plantlets produced much shorter functional roots whose overall structuration suggests both activated cell division and cell differentiation rates in the transgenic root tips. In the epidermis of S plantlets, the differentiation of root hairs could be noticed in the near vicinity of the RAM (Figure 4e). Such ectopic cell differentiation patterns affecting the epidermis layer were also be observed in the upper part of the transgenic roots where adjacent trichoblasts produce root hairs (Figure 4h). Compared to the observations previously reported in transgenic *Arabidopsis* [22], the S and AS phenotypes we observe could certainly result from stronger overexpression and inactivation of *AtAGL12* due to the use of the d35S promoter. Indeed, the upper parts of transgenic S roots obtained with this stronger promoter are also characterized by the development of ectopic cell masses (Figure 4h) that share important morphological and developmental similarities with the abnormal “root primordial masses” (RPMs) induced in pea seedlings roots upon cultivation with different auxin transport inhibitors [41,42]. In S plantlets roots, the formation of such RPMs lacking complete apical differentiation appears to be linked to the activation of pericycle cells as a consequence of *AtAGL12* overexpression. In wild-type plants when lateral roots are normally formed, only a few pericycle cells located close to the vascular xylem poles are activated. These activated xylem pole pericycle cells undergo first periclinal and then anticlinal divisions to form small lateral root primordia that are regularly separated due to an oscillatory gene expression system [43]. The same kind of development can initially be observed in the RPMs developing in S roots (Figure 4i, arrows) and in treated pea roots [41]. In both cases, relatively larger rows of pericycle cells adjacent to xylem vessels appear to be activated resulting in enlarged responsive areas. Anticlinal and periclinal divisions would then lead to the thickening of these structures that could correspond to abnormally enlarged or merging lateral root primordia expanding around the stele. The use of reporter constructs driven by xylem pole pericycle cell and lateral root primordia specific promoters could allow to verify this hypothesis [44,45].

In wild-type *Arabidopsis* root, the expression of *AGL12/Xal1* has been reported in the primary root meristem, the central cylinder, the lateral root primordia originating from the pericycle and the atrichoblasts within the epidermis [19,20]. In the present study, one can notice that the overexpression of *AtAGL12* also appears to affect each of these root tissues including the epidermis (Figure 4h). So far, *AGL12* is thought to be an upstream regulator of *SOC*, *FLOWERING LOCUS T* and *LFY* during *Arabidopsis* flower induction while involved in the regulation of cell divisions and secondary cell wall synthesis in the root [20,21,22]. In *C. roseus*, its overexpression affected cell division and aggregation as well as alkaloid production [36]. Its involvement in these different aspects of plant physiology places this TF at the crossroad of different pathways that remain to be elucidated in terms of interacting partners as well as downstream targets. In *Arabidopsis*, the results of large scale two-hybrid yeast experimentations superposed to microarray expression patterns highlighted specific interactions between MADS-box proteins marked by the formation of tetrameric transcription regulatory units in various tissues [46,47]. According to these data, *AGL12* could interact with *AGL21* and/or *AGL16* to regulate different downstream processes in roots such as meristem cell division and differentiation, secondary cell-wall synthesis, secondary metabolite production and/or lateral root initiation. Thus, *AGL12* and other MADS-box TFs could lie upstream of or interfere with various GRN regulating the specific differentiation process that are being established in roots [30,48,49,50,51].

In conclusion, our results confirm the prominent role of *AGL12* in root meristem cell division and differentiation processes in *Juglans sp.* and *Arabidopsis*. Transcriptomic analysis of the genes affected by *AGL12* misregulation should provide us with a clearer view of its role during root development and allow us to determine the corresponding GRN linked to the drastic phenotypic alterations we have obtained. *AGL12* and other “root-specific” MADS-box genes could represent interesting candidates to engineer root development or root architecture in plants, rootstocks or trees. Beyond any biotechnological aspects concerning the production and the use of genetically modified organisms, discovering the GRN linked to root development through the analysis of master genes like *AGL12* in GM trees should lead to the determination of yet unexplored genetic markers linked to their root foraging abilities. Until now, tree breeding programs have mainly been based on the selection of apical traits (e.g., disease resistance, trunk growth and straightness, branching, wood quality, bud set…). In fine, the characterization of such genetic markers in *Populus* or *Eucalyptus sp*. might be of great interest for carbon allocation, wood biomass production and drought resistance studies in these tree models.

## 4. Materials and Methods

### 4.1. Plant Materials

*Juglans nigra* x *J. regia* somatic embryos (line I1C) maintained through repetitive secondary embryogenesis were sub-cultured every 3 to 5 weeks on hormone- and L-glutamine-free DKW medium as previously described [52,53]. *Arabidopsis thaliana* (Col-0) were cultured in peat pots under standard greenhouse conditions (23 °C, photoperiod 16 h, regular watering).

### 4.2. Binary Vector Construction

*Arabidopsis thaliana AGL12* cDNA (clone *pSR102*, accession U20193) and promoter sequence (clone *pSR212* derived from Clontech *pBI101.1*) were kindly provided by Prof. Martin Yanofsky (U.C. San Diego). *AtAGL12* full-length cDNA sequence was excised from the *PCR 2.1* vector (Invitrogen) by *Eco*RI digestion and inserted into *pLBR19* under the control of the double 35S Cauliflower Mosaic Virus promoter [23,25]. Expression cassettes containing *AtAGL12* cDNA in sense (S) or antisense (AS) orientation were selected through asymmetric *Xba*I restriction digestion and transferred into the *pBINPLUS* binary vector carrying *nptII* sequence conferring kanamycin resistance [54]. Binary plasmids with *AtAGL12* promoter::*GUS* (*pSR212*) and d35S::*GUS-intron* (*pKYGIN*) fusions were added to the study. The four constructs (*d35S::AtAGL12S*, *d35S::AtAGL12AS*, *pAtAGL12::GUS* and *d35S::GUS-intron*) were then transferred by triparental mating into *Agrobacterium* (C58/pMP90).

### 4.3. Plant Transformation

Genetic transformation of walnut tree I1C somatic embryos was performed according to [25] with the following modifications. For each *Agrobacterium* strain, 50 to 75 white cotyledonary embryos (3–5 mm) were immersed for 2 h in 25 mL of bacterial suspension (2.5 × 10^8^ cfu/mL, 200 rpm) in the presence of acetosyringone (100 μM) and sterilized Fontainebleau sand (2 g). The embryos were then recovered and plated onto solid DKW medium supplemented with acetosyringone (100 μM). After 48 h the embryos were collected, rinsed 3 times in 50 mL of liquid DKW medium (20 min, 200 rpm) supplemented with cefotaxim (500 mg/L) and ticarpen (250 mg/L), and placed on solid DKW medium supplemented with the same antibiotics. After one week, the embryos were transferred onto solid DKW medium only supplemented with kanamycin (500 mg/L). After 4 subcultures (15 days each) in the presence of kanamycin, emerging secondary embryos were sampled and individually sub-cultured 5 more times on kanamycin-supplemented medium to establish kanamycin resistant (kan^R^) embryonic lines. Preliminary molecular screens, gus staining and/or phenotypic observations were then performed to confirm their transgenic status and coarsely evaluate their spontaneous germination abilities. At the end of this selection process, 10 to 12 transgenic lines obtained from each construct (*d35S::AtAGL12S*, *d35S::AtAGL12AS*, *pAtAGL12::GUS*, and *d35S::GUS-intron*) were selected, and thereafter routinely cultivated only on antibiotic-free medium, and further referred in the text as S, AS, PAG, and GIN lines, respectively.

*Arabidopsis thaliana* transformations were realized through the “floral-dip” method [55]. Seeds obtained from treated inflorescences were surface sterilized, imbibed for 48 h at 4 °C and sown on MS medium [56] supplemented with kanamycin (100 mg/mL) in a growth chamber (16 h photoperiod, 200 μmol photons m/s, 15 °C/20 °C night/day, 70% relative humidity). Two days after germination (DAG), green kan^R^ T_1_ plantlets were selected, transferred on the same medium lacking the selective antibiotic for phenotypic observations and transferred to the greenhouse to produce T_2_ seeds. Imbibed WT seeds were directly sown on kanamycin free medium.

### 4.4. Molecular Biology

Germinated and mature non-germinated somatic embryos (250 mg) were sampled, frozen in liquid nitrogen, lyophilized, and used for DNA and RNA extractions [53]. RNA obtained from germinated wild-type (I1C line) somatic embryos were used to clone a partial walnut *AGL12* cDNA sequence by 3′ RACE-PCR using the same strategy as previously described [53]. Its sequence (*wAGL12*, accession number MF327581) is presented in Supplemental Figure S1.

The transgenic status of the kan^R^ walnut tree embryonic lines was confirmed by at least two of the following means: gus staining, PCR, RT-PCR, Southern-dot blot or Southern-blot (Supplemental Table S1) during the kanamycin selection process (GIN and PAG lines) or after a preliminary screen of their germination abilities (S and AS lines). Briefly, T-DNA insertions within PAG, S and AS kan^R^ lines were primarily confirmed by means of PCR amplifications using different combination of primers. Then, complementary Southern-dot- and Southern- blot analysis performed with a *nptII* probe on selected PCR+ lines were used to detect transgene presence. Southern-dot blot membranes used for large scale molecular screenings of kan^R^ lines were realized with 1 μg of denatured DNA using a 96 well microfiltration unit (Bio-Dot, Bio-RAD). Southern-blots were then performed with 5 μg of digested DNA on a fewer number of lines to estimate transgene insertion events. Hybridization and washing conditions of these membranes were the same as those used for northern-blot analysis (see below). For transgene expression analysis, northern-blots were realized with 20 μg of total RNA obtained from mature non-germinated S somatic embryos. Membranes were probed with amplified cDNA fragments of *AtAGL12* and *Juglans* Rib60S (accession AJ278460), washed twice in 0.2x SSC, 2% w/v SDS at 65 °C before autoradiography [53].

### 4.5. Histochemical Revelation of B-glucuronidase Activity

Explants of walnut tree somatic embryos and embryonic roots (GIN and PAG lines) were vacuum infiltrated with 0.1 M phosphate buffer (pH 7.0) containing X-GlcA (1 mM, B5285 Sigma), Na_2_EDTA (10 mM), potassium ferricyanide (1 mM), potassium ferrocyanide (1 mM), methyl hydrate (20% v/v), and Triton X-100 (0.5% v/v). After 3 to 16 h of incubation at 37 °C, the explants were rinsed, fixed in 70% ethanol, and observed under a Nikon SMZ1000 stereomicroscope equipped with a Nikon DS-Fi1 digital camera.

### 4.6. Root Development Analysis

In *Juglans sp.*, spontaneous in vitro germination abilities and root development characteristics of somatic embryos were determined for 10 representative transgenic S lines that presented upon preliminary screening: strikingly higher, higher or equivalent spontaneous germination rates compared to the original I1C line. GIN, PAG, and AS embryonic lines were added to the phenotypic analysis as controls. For each line, 3 replicates of 10 embryos (2–5 mm, early cotyledonary stage) were placed on solidified hormone -, kanamycin -free DKW medium in 70 mm Petri dishes (dark, 27 °C). After 3 weeks, the developing cotyledons were ablated and the embryonic axis aligned on the same medium in plates (120 × 120 mm) placed vertically for 2 additional weeks. Primary and lateral root appearances were scored at the end of each phase. Rooted embryos were then individually transferred in culture tubes (16 h photoperiod). After 5 weeks, the root and shoot apices of each plantlet were separated, lyophilized, and weighted on a precision balance (Pioneer P114C, Ohaus corp.). These experiments were repeated twice and statistical analyses were performed using R software.

In *Arabidopsis*, phenotypic observations were performed on at least 5 in vitro germinated T_1_ kan^R^ S and AS plantlets grown for one to three weeks on kanamycin free medium. WT plantlets were grown in parallel on kanamycin free medium. Due to the important differences observed between S, AS and WT *Arabidopsis* plantlets, only three S T_1_ and WT individuals were sampled for histological observations. One and two of these plants were respectively used for transversal and longitudinal sections of their roots. Every other AS and S T_1_ available were transferred to the greenhouse to produce T_2_ seeds. T_2_ seeds and plantlets were treated as T_1_.

### 4.7. Microscopic Analysis

All plant tissue samples were fixed with 2.5% glutaraldehyde in 0.1M pH 6.8 citrate phosphate buffer for 4 h at room temperature. Explants were then post-fixed in osmium tetroxyde 1% (w/v), dehydrated through an ethanol series, and embedded in epoxy resin (EPON). Longitudinal and transversal semi-thin sections (1 µm) stained with azure II/methylene blue were observed with a light microscope (Leica-DMR) and photographed. Vascular tissue organization (cell counts) of *Arabidopsis* roots were performed on median longitudinal sections of their root tips and on two perpendicular diagonals on transverse sections (Supplemental Figure S2).

## Figures and Tables

**Table 1:** Walnut tree transformation and transgenic line selection. The kanamycin resistant (Kan^R^) *Juglans* sp. embryogenic lines growing after 5 subcultures on selective medium were further analyzed by molecular means to confirm their transgenic status. Only the lines showing two positive PCR amplifications of different T-DNA targets (*nptII* and *GUS* or *AtAGL12*) from their genomic DNA were further considered (PCR^+^ lines). When relevant, the numbers of line showing positive GUS staining are given (GUS^+^). In the end, the transgenic lines selected for phenotypic analysis are listed.

Transformation Constructs	Kan^R^ Line Number	PCR^+^ Line Number	GUS^+^ Line Number	Transgenic Lines Selected for Phenotypic Analysis
***d35S::GUS Intron***	60	27 (45%)	26 (96%)	GIN: 1, 4, 5
***pAtAGL12::GUS***	70	27 (38%)	15 (55%)	PAG: 1, 15, 16, 46, 48, 75
***d35S::AtAGL12S***	109	45 (41%)	NA ^1^	S: 53, 55, 59, 64, 69, 76, 77, 101, 105, 109
***d35S::AtAGL12AS***	59	30 (51%)	NA ^1^	AS: 20, 27, 29, 45

^1^ Not applicable.

**Table 2:** Transgenic walnut plantlet development. Leaf numbers, lengths, root and shoot dry weights and root/shoot ratios (R/S) were obtained for each plantlet. For statistical analysis, the data were gathered according to the types of line they originated from: S (S lines), WT (I1C wild-type) and other types of transgenic lines (GIN, PAG and AS). The total number of plantlets analyzed for each type of line is indicated (n).

Type of Line	n	Leaf Number	Stem Length (mm)	Root length (mm)	R/S (length)	Stem dw (mg)	Root dw (mg)	R/S (dw)
**S**	19	1.6 ± 0.9 *	9.5 ± 1.6 *	53.8 ± 11.1*	6.8 ± 1.9 *	5.7 ± 1.4 *	52.6 ± 12.7 *	12.0 ± 4.1 *
**WT**	20	3.0 ± 0.9	16.7 ± 5.1	30.6 ± 7.6	2.8 ± 1.1	11.4 ± 3.5	30.9 ± 8.9	3.6 ± 1.0
**AS**	23	1.9 ± 0.9	11.7 ± 4.4	39.0 ± 8.5	4.8 ± 1.4 *	10.4 ± 4.2	45.0 ± 11.7	7.2 ± 3.6
**GIN**	8	1.0 ± 1.1 *	8.6 ± 2.0	58.3 ± 18.0 *	6.9 ± 1.7 *	7.8 ± 2.5	54.4 ± 16.5 *	9.4 ± 6.2 *
**PAG**	13	2.2 ± 1.2	16.5 ± 6.2	65.2 ± 17.3 *	8.6 ± 6.3 *	12.2 ± 4.3	59.2 ± 15.2 *	8.4 ± 6.3

* significantly different from non-transgenic I1C (Student’s *t* test, *p* < 0.05).

## Supplementary Materials

The following are available online at https://www.mdpi.com/2223-7747/9/4/444/s1, Figure S1: Sequence alignment of walnut and *Arabidopsis* AGL12 cDNA. Figure S2: Transverse sections of WT (Col-0) and S T_1_ *Arabidopsis* roots sampled 3 weeks after germination. Table S1: List of primers and methods used for transgene detection in walnut.
