# Supplementary material for: Overexpression of MADS-box Gene AGAMOUS-LIKE 12 Activates Root Development in Juglans sp. and Arabidopsis thaliana
Source: Plants (Basel). 2020 Apr 2;9(4):444. doi: 10.3390/plants9040444 (PMC7238194; doi:10.3390/plants9040444)
Supplement: Supplementary file 1 [file plants-09-00444-s001.pdf]

plants

MDPI

```
AtAGL12         AAATCATCAGATAGAAGGAAATATTCTGATTGAGAGATGGCTCGTGGAAAGATTCAGCTT
wAGL12          ------------------------------------------------------------

AtAGL12         AAGAGGATTGAGAACCCGGTTCACAGACAAGTGACTTTTTGCAAGAGGAGAACTGGTCTT
wAGL12          ------------------------------------------------------------

AtAGL12         CTCAAGAAGGCTAAGGAGCTCTCTGTGCTCTGTGATGCCGAGATCGGTGTTGTGATCTTC
wAGL12          --TAAGAAGGCCAAGGAGCTTTCTGTGCTGTGTGATGCTGAAATTGGAGTTATCATTTTC
                   ******** ******** ******** ******** ** ** ** *** * ** ***

AtAGL12         TCTCCTCAGGGCAAGCTCTTTGAGCTCGCTACTAAAGGAACAATGGAGGGAATGATTGAT
wAGL12          TCCTCCCATGGAAAGCTCTATGAGCTGGCCACCAAAGGAACCATGCAAGGGATTACTGAG
                **  * ** ** ******* ****** ** ** ******** *** * ** ** * ***

AtAGL12         AAGTACATGAAGTGTACTGGTGGTGGTCGTGGTTCTTCTTCTGCTACTTTTACTGCTCAA
wAGL12          AGGTACATGAAGTCTACAGGAGAGGTTCAGCCTGAACC---------------------A
                * *********** *** ** *  * **    *    *                     *

AtAGL12         GAACAACTTCAACCACCAAATCTTGATCCGAAAGATGAGATCAACGTGCTTAAGCAAGAG
wAGL12          GCCATTGAAGCACAACCCTGCCTGGATGCAAAAGAGGAAATTAACATGCTGAAACAAGAG
                *          ** ***    ** *** * ***** ** ** *** **** ** ******

AtAGL12         ATTGAGATGCTTCAGAAAGGGATAAGCTATATGTTTGGAGGAGGAGATGGGGCTATGAAT
wAGL12          ATCGACATACTCCAAAAAGGTCTCAGGTATATGTTTGGAGGTGGAGGTGCGACAATGACG
                ** ** ** ** ** *****  * ** ************** **** ** * * ****

AtAGL12         CTTGAAGAACTTCTTTTGCTTGAGAAGCATCTTGAGTATTGGATTTCTCAGATTCGCTCT
wAGL12          TTGGATGAGTTAGATGTGCTTGAAAAGCACCTTGAGGTTTGGATTTGTAACATACGTTCA
                 * ** **  *   * ******* ***** ******  ******** * * ** ** **

AtAGL12         GCTAAGATGGATGTTATGCTTCAAGAAATTCAGTCATTGAGGAACAAGGAAGGAGTCCTC
wAGL12          ACAAAGATGAACATTATGTTTCAAGAGATTCAACTTTTGAGGAATAAGGAAGGAATGCTG
                 * ****** *  ***** ******* *****    ******** ********* * **

AtAGL12         AAAAACACCAACAAGTATCTCCTCGACAAGATAGAGGAAAACAACAATAGCATATTAGAT
wAGL12          AAAGCTGCAAATAAGTATCTCCAAGATAAGATAGAAGATCATCAGAACAGTAGTACTACT
                ***    * ** **********  ** ******** **  *  * ** ** *       *

AtAGL12         GCTAACT------TCGCAGTCATGGAGACAAACTATT---CCTATCCGCTAA---CAATG
wAGL12          GCAATCACTGACTTCGCACCAATTAATACCACTAATTTCCCATACCCACTAACCATAATG
                ** * *       *****   **  * ** *   ***   * ** ** ****    ****

AtAGL12         CCAAGTGAAATATTTCAGTTCTAGACCATAGGGTATTTGAAGACTATGTCTCACGAATTT
wAGL12          CAGAATGAGATATTGAAATTCTAGCTAGGATTGTCAT-----------------GATCTA
                *  * *** *****  * ******     *  **  *                 **  *

AtAGL12         AAATAACCTTGGTAAGT-----ATAATATAGTGTTGTTAAATCAC---ACATAAT-----
wAGL12          GAATAAGCTGATCTACTGTTTTATGTACTACTGTTTTTAATTAAGGTCACGCGCGTGTTC
                 ***** **     * *     **    ** **** **** * *    **

AtAGL12         ---TAAAATAAAGCCTGTGGAACTTCGCTAGGCAGTTGAA-----AATCTATCCGTATGT
wAGL12          TGTTCTCATGTAAGCCAATGTACGTAGCTGTATAGTATAAATTAAGTTCTTTCCGATGGA
                   *   **  *  *    * ** * ***    ***  **       *** ****   *

AtAGL12         TTTATCCTCTTGTTTTACATTTGTTGGTGTGAAGATGAAA---T-GACTGCAAGTGTGGT
wAGL12          CAGACGTACGTACGTT----GTAATTGTCTGATCATGATGATCAGGATGGGTACTTGCCA
                   *    * *   **     *  * ** ***  ****       **  *  * *

AtAGL12         GTGTACTTATAACTCTTTCTACTTTCTATCTATGTTTTGAATTTATGGATT---------
wAGL12          TCCTAGCTAGTACTCTTACTACTTTAGCAGCTTGATTTGTAAGTATATTAATGTTGACGT
                   **  **  ****** *******       ** **** *  ***

AtAGL12         ------------------------------------------------------------
wAGL12          ATTGTGGTCTTGTTTGCTATCAATAATGCTTGCATATATGCGTGTCATGTTATTCAAAAA

AtAGL12         --------------------
wAGL12          AAAAAAAAAAAAAAAAAAAA
```

**Figure S1.** Sequence alignment of walnut and *Arabidopsis AGL12* cDNA. The partial sequence of a walnut *AGL12* (*wAGL12*, accession number MF327581) cDNA was cloned by RT-PCR according to [50] and aligned with the *AtAGL12* cDNA sequence used for transformation. Briefly, after amplification and sequencing of the MADS-box sequences expressed in walnut roots, the 3′ end of the cDNA were amplified with the following gene specific primer (5′TAAGAAGGCCAAGGAGCTTT3′). The PCR products were cloned, sequenced and aligned with *AtAGL12* cDNA using Clustal Omega revealing 62.8% nucleotide identity (*) between both sequences. Translation initiation and termination codon are in bold characters.

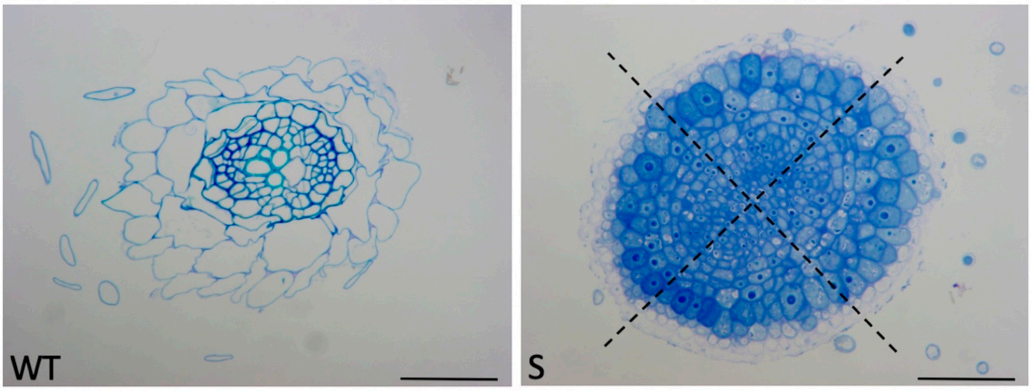


**Figure S2.** Transverse sections of WT (Col-0) and S $T_1$ *Arabidopsis* roots sampled 3 weeks after germination. The dotted lines represent two examples of perpendicular diagonals drawn for cell counting on root cross sections (equivalent to median planes on longitudinal sections, Figure 4). Scale bars: 100µm.

**Table S1.** List of primers and methods used for transgene detection in walnut. The table summarize the primers used to characterize the walnut tree transgenic lines and produce cDNA probes (*AtAGL12, gus and nptII)*. Kan$^R$ embryonic lines were first screened by PCR and Southern-dot blot and further characterized by Southern- and/or northern blot hybridizations (mentioned in the last column of the table (P, Sd, S, and N, respectively).

| Primer | Primer Sequence (5′-3′) | Annealing Temp. (°C) | Amplified Sequence (pb) | Method |
|---|---|---|---|---|
| **d35S** | gacgcacaatcccactatcc | 60 | S an AS constructs | P |
| **agl12s** | tctctgtgctctgtgatgcc | | | |
| **agl12as** | ttcacttggcattgttagcg | 60 | *AtAGL12* (500) | P, Sd, N |
| **nptIIa** | tgttccggctgtcagcgcag | | | |
| **nptIIb** | tcggcaagcaggcatcgcca | 60 | nptII (477) | P, Sd, S |
| **gusa** | tatacgccatttgaagccg | | | |
| **gusb** | aagccagtaaagtagaacggt | 60 | gus (550) | P, Sd |
| **mcs1** | ccaggctttacactttatgc | | | |
| **mcs2** | tcacgggttggggtttctac | 50 | *AtAGL12* promoter (>3000) | P |
| **agrA** | ccgtttccatttcgtcatatttc | | | |
| **agrB** | taaccgtgaacgtatagaccaccag | 60 | *Agrobacterium gDNA (560)* | P |
